# Abdominal Ultrasound Detection of Portal Vein Gas in Non-occlusive Mesenteric Ischemia: A Case Report

**DOI:** 10.7759/cureus.94305

**Published:** 2025-10-10

**Authors:** Hideya Itagaki, Katsuhiko Suzuki

**Affiliations:** 1 Emergency and Disaster Medicine, Tohoku Medical and Pharmaceutical University Hospital, Miyagi, JPN; 2 General Surgery, Honjou Daiichi Hospital, Yurihonjou, JPN

**Keywords:** abdominal ultrasound, computed tomography, intestinal necrosis, non-occlusive mesenteric ischemia, portal vein gas, ultrasonography

## Abstract

Portal vein gas (PVG) is often associated with severe gastrointestinal disease and has traditionally been considered a marker of poor prognosis. While computed tomography (CT) is the most common modality for PVG detection, reports of detection via abdominal ultrasound (US) are limited. A 91-year-old bedridden woman presented with diarrhea and hematochezia. Abdominal US revealed multiple hyperechoic foci within the liver consistent with PVG, whereas CT demonstrated only a few PVG spots and non-enhancing small bowel segments. In the absence of superior mesenteric artery occlusion, she was diagnosed with non-occlusive mesenteric ischemia (NOMI). Emergency laparotomy revealed a 15-20 cm necrotic ileal segment, which was resected with formation of a double-barrel ileostomy. Histopathology confirmed NOMI. Postoperative recovery was uneventful, and she was discharged on day 19. Follow-up US within 24 hours of surgery showed complete resolution of PVG. This case demonstrates that the US may detect PVG more sensitively than CT in certain situations. Early US evaluation could facilitate timely diagnosis and intervention in severe abdominal conditions and may provide prognostic information based on the sonographic PVG pattern.

## Introduction

Portal vein gas (PVG) is an uncommon radiological finding most often associated with intestinal ischemia, necrosis, inflammatory bowel disease, or intra-abdominal abscess [1,2]. Historically, PVG has been associated with mortality rates of up to 75%, although advances in computed tomography (CT) technology have reduced this to approximately 29-39% [3,4]. CT remains the standard imaging modality for detecting PVG; however, ultrasound (US) can also visualize gas within the portal venous system and offers advantages such as bedside availability and real-time assessment [5]. Comparative reports of PVG detection by US versus CT remain limited. Non-occlusive mesenteric ischemia (NOMI), first described by Ende in 1958, is characterized by segmental or discontinuous mesenteric ischemia and intestinal necrosis without an organic obstruction in the mesenteric vessels [6]. Diagnosis of NOMI usually relies on angiography or multidetector-row CT, whereas the utility of US for diagnosing this condition remains uncertain [6]. We present a case of NOMI in which PVG, showing a fruit-pulp-like pattern, was more conspicuous on US than on CT, highlighting the potential role of US in early detection and prognostic assessment. The patient presented with diarrhea and hematochezia, accompanied by fever and tachycardia, which prompted further imaging evaluation for suspected intestinal ischemia.

## Case presentation

A 91-year-old bedridden woman presented to the emergency department at Honjo Daiichi Hospital with diarrhea and hematochezia. She reported the onset of frequent diarrhea the previous afternoon, progressing to hematochezia the following morning. Her medical history included hypertension and diabetes mellitus, with no family history of gastrointestinal malignancy or inflammatory bowel disease.

On arrival, the patient was drowsy, with a blood pressure of 144/51 mmHg, a heart rate of 118 beats per minute, a body temperature of 38.0°C, an oxygen saturation of 98% on room air, and a respiratory rate of 25 breaths per minute. She exhibited fever, tachycardia, and tachypnea. Abdominal examination revealed a soft but distended abdomen with diffuse tenderness on palpation. Laboratory tests revealed an elevated C-reactive protein level (Table 1).

**Table 1:** Blood tests at admission WBC, white blood cell count; RBC, red blood cell count; Hb, hemoglobin; PLT, platelet count; TP, total protein; AST, aspartate aminotransferase; ALT, alanine aminotransferase; ALP, alkaline phosphatase; LDH, lactate dehydrogenase; BUN, blood urea nitrogen; Cre, creatinine; Na, sodium; K, potassium; CRP, C-reactive protein

Complete Blood Count Data		
Parameter	Result	Reference value
WBC	6.8	3.3-8.6 (×10^3^/μL）
RBC	4.8	4.35-5.55 (×10^6^/μL）
Hb	14.5	13.7-16.8 (g/dL）
Plat	174	158-348 (×10^3^/μL）
Biochemistry Data		
Parameter	Result	Reference value
TP	6.3	6.6-8.1 (g/dL）
AST	17	13-30 (U/L）
ALT	10	10-42 (U/L）
ALP	66	38-113 (U/L）
LDH	223	124-222 (U/L）
BUN	27.4	8-20 (mg/dL）
Cre	0.83	0.65-1.07 (mg/dL）
Na	141	138-145 (mmol/L）
K	4.1	3.6-4.8 (mmol/L）
CRP	6.88	0.0-0.14 (mg/dL）

**Figure 1:**
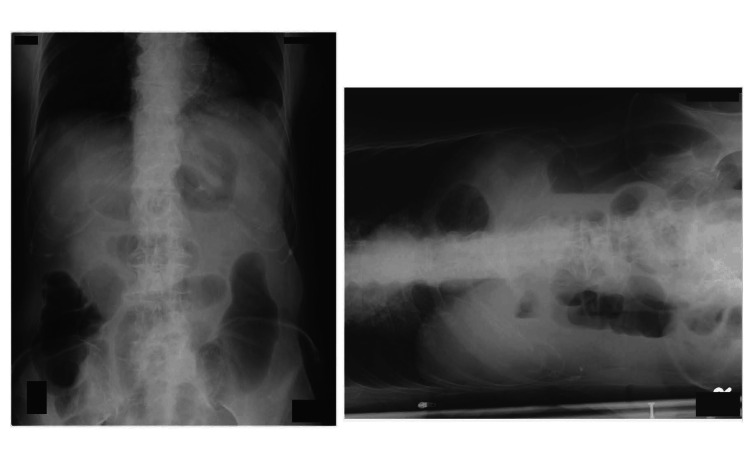
Bowel gas on X-ray Supine (left) and side-lying (right) abdominal radiographs. The side-lying position was used instead of the upright position to evaluate for air-fluid levels because the patient was unable to stand.

Plain abdominal radiography showed multiple air-fluid levels (Figure 1).

**Figure 2:**
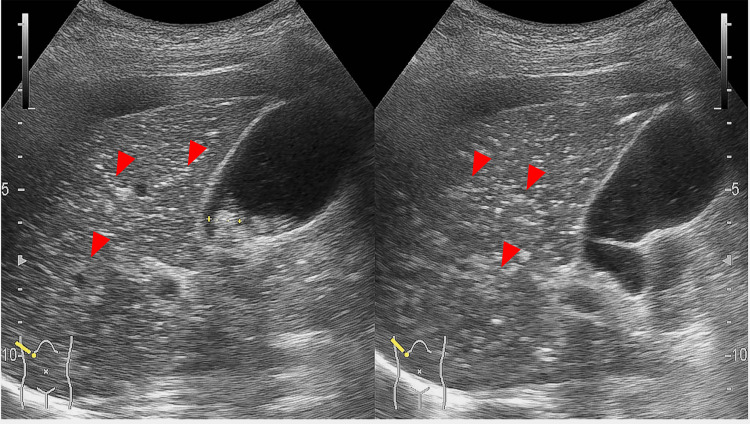
Abdominal ultrasound detection of portal vein gas Abdominal ultrasound image of a 91-year-old woman with non-occlusive mesenteric ischemia. Multiple hyperechoic foci within the liver parenchyma (red arrowheads) are more conspicuous than on computed tomography, consistent with portal vein gas. Numerous hyperechoic foci are distributed throughout both hepatic lobes, extending to within approximately 1 mm of the liver capsule: “fruit-pulp-like” pattern.

Abdominal US was performed to evaluate for bowel obstruction and revealed multiple hyperechoic foci scattered within the liver parenchyma, consistent with PVG (Figure 2), as well as bowel dilatation in the left lower quadrant.

**Figure 3:**
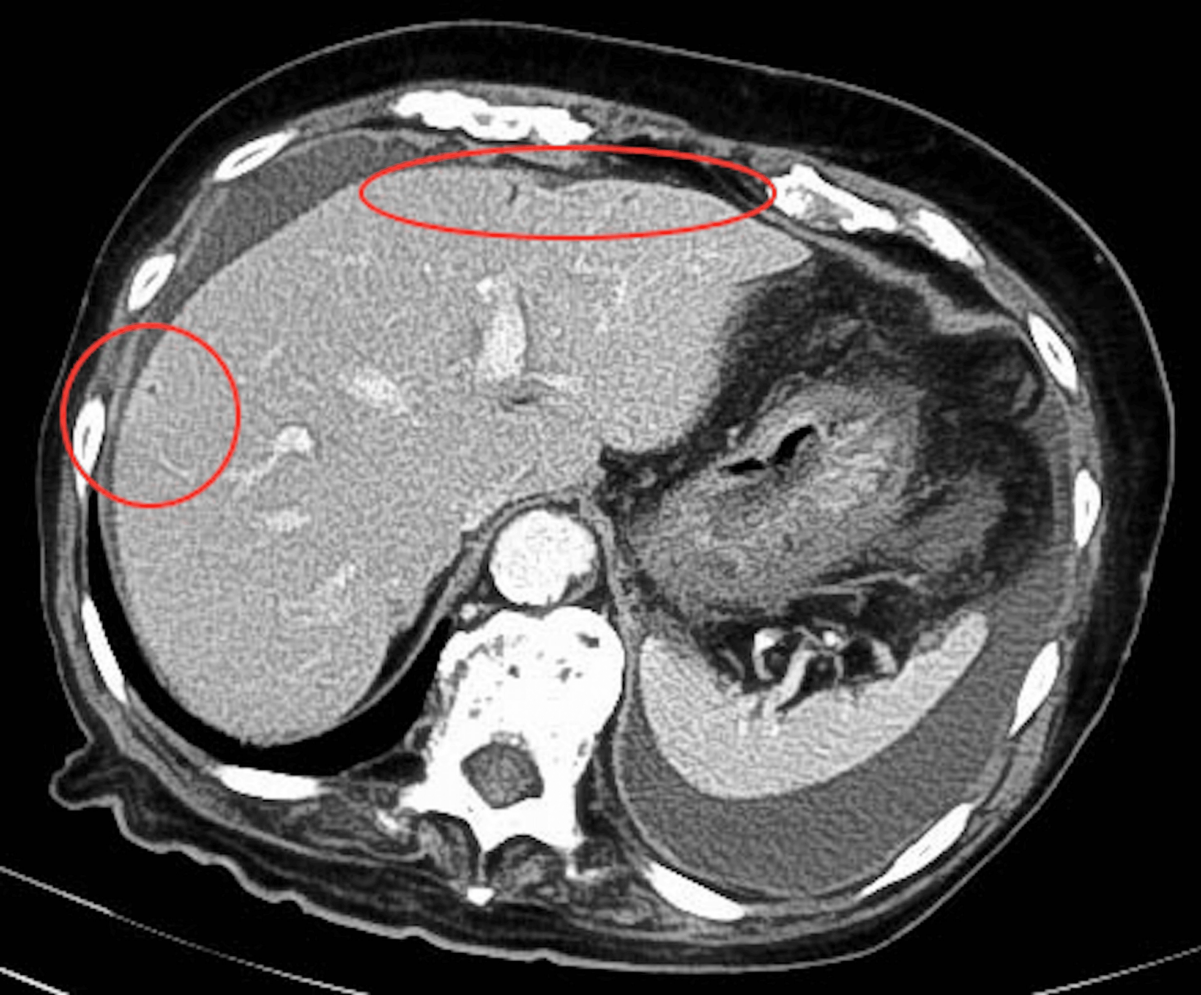
Computed tomography findings of portal vein gas Contrast-enhanced computed tomography of the upper abdomen demonstrates only a few small gas foci in the portal vein (red circle), less evident than on ultrasound, in the same patient.

Contrast-enhanced CT showed only a few PVG spots (Figure 3) and non-enhancing small bowel loops, with no evidence of superior mesenteric artery occlusion. Based on these findings, a diagnosis of NOMI was made.

**Figure 4:**
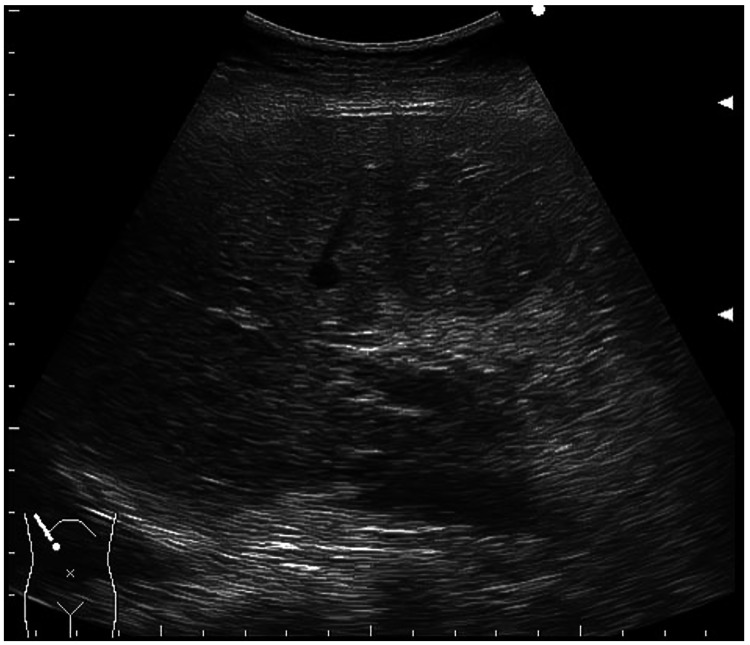
Resolution of portal vein gas after surgery Follow-up abdominal ultrasound performed within 24 hours after resection of necrotic ileum showed the complete disappearance of portal vein gas within the liver parenchyma.

Emergency laparotomy revealed a 15-20 cm necrotic ileal segment near the ileocecal junction. Resection was performed, and a double-barrel ileostomy was created to minimize the risk of anastomotic leakage given the patient’s frailty. Histopathology confirmed NOMI. The patient received postoperative antibiotics and vasodilator therapy. Doripenem 0.25 g was administered intravenously three times daily, and nicorandil was given at 48 mg/day for one week as vasodilator therapy. Her recovery was uneventful, and she was discharged on postoperative day 19. Follow-up US within 24 hours post-surgery showed complete disappearance of PVG (Figure 4). 

## Discussion

PVG is associated with various clinical conditions, including intestinal necrosis, inflammatory bowel disease, and intra-peritoneal abscess, and carries a reported mortality rate of up to 75% [1]. Advances in CT have enabled earlier detection, reducing mortality to approximately 29-39% [4,5]. Nonetheless, the presence of PVG remains an indicator of severe underlying disease.

In our patient, both abdominal US and CT were used to evaluate suspected intestinal ischemia. PVG was more conspicuous on US than on CT, consistent with prior reports in which PVG was detected by US but not by CT [7,8]. This highlights the potential for the US to be more sensitive in identifying PVG.

The temporal course of PVG in this case was similar to that described in a previous NOMI report, where US performed nine hours after surgery showed a transient reduction in PVG and complete disappearance after the patient had stabilized [9]. In our case, PVG resolved within 24 hours of necrotic bowel resection, supporting the notion that PVG can be detected during the acute phase and disappears rapidly after the underlying cause is treated.

Pan et al. classified sonographic PVG into three patterns, dot-like, streak-like, and fruit-pulp-like, based on the extent and distribution of echogenic foci in the liver parenchyma [10]. Dot-like PVG represents a small gas volume, is often transient, may be absent on CT, and generally carries a favorable prognosis. In contrast, streak-like and fruit-pulp-like patterns indicate a larger gas burden, are usually visible on both US and CT, and, when unrelated to localized liver lesions, are frequently associated with life-threatening bowel ischemia and poor outcomes [10].

Our patient’s PVG exhibited a fruit-pulp-like pattern on US, extending to within 1 mm of the liver capsule, in keeping with Pan et al.’s description of a high-risk pattern. CT performed shortly after US showed only scant PVG, underscoring the superiority of US in detecting small intraportal gas bubbles. This superiority is attributed to the US’s real-time visualization of mobile echogenic particles, the high acoustic impedance contrast of gas against a dark background, and the partial-volume effect that can limit CT detection. Notably, another study comparing CT and US found that we confirmed PVG but not CT in one of two survivors among seven patients diagnosed with portomesenteric venous gas [11], further supporting the value of US in both early detection and potential prognostic assessment.

Although the US has several advantages, such as bedside availability and real-time assessment, it also has notable limitations compared with CT. The US is operator-dependent, may be affected by bowel gas, and cannot fully evaluate mesenteric perfusion or vascular anatomy [12]. CT angiography remains the current gold standard for diagnosing mesenteric ischemia because it provides comprehensive information on vessel patency and bowel viability [13]. In the present case, CT angiography was not performed due to institutional limitations; our facility did not have the technical capability to perform CT angiography at that time. Nevertheless, the combination of conventional contrast-enhanced CT and US provided sufficient diagnostic information to guide appropriate surgical management.

Overall, this case demonstrates that the US can not only detect PVG earlier than CT but may also provide prognostic insights based on the observed sonographic pattern. Further research is needed to confirm whether early identification of high-risk PVG patterns in the US can enhance clinical decision-making and patient outcomes.

## Conclusions

Abdominal US demonstrated PVG more conspicuously than CT in this single case of NOMI with a fruit-pulp-like PVG pattern. Early US assessment in patients with suspected mesenteric ischemia may provide practical bedside value and could potentially yield prognostic insights based on sonographic PVG patterns. However, given the limitations of a single case, these observations should be regarded as hypothesis-generating. Further studies are warranted to clarify the prognostic significance of specific US patterns and to determine whether US might serve as a helpful adjunct, rather than a replacement, to CT in this clinical context.
